# Improved Cross-Ratio Invariant-Based Intrinsic Calibration of A Hyperspectral Line-Scan Camera

**DOI:** 10.3390/s18061885

**Published:** 2018-06-08

**Authors:** Daobilige Su, Asher Bender, Salah Sukkarieh

**Affiliations:** Australian Centre for Filed Robotics (ACFR), The University of Sydney, Sydney, NSW 2006, Australia; a.bender@acfr.usyd.edu.au (A.B.); s.sukkarieh@acfr.usyd.edu.au (S.S.)

**Keywords:** camera calibration, line-scan camera, hyperspectral camera

## Abstract

Hyperspectral line-scan cameras are increasingly being deployed on mobile platforms operating in unstructured environments. To generate geometrically accurate hyperspectral composites, the intrinsic parameters of these cameras must be resolved. This article describes a method for determining the intrinsic parameters of a hyperspectral line-scan camera. The proposed method is based on a cross-ratio invariant calibration routine and is able to estimate the focal length, principal point, and radial distortion parameters in a hyperspectral line-scan camera. Compared to previous methods that use similar calibration targets, our approach extends the camera model to include radial distortion. It is able to utilize calibration data recorded from multiple camera view angles by optimizing the re-projection error of all calibration data jointly. The proposed method also includes an additional signal processing step that automatically detects calibration points in hyperspectral imagery of the calibration target. These contributions result in accurate estimates of the intrinsic parameters with minimal supervision. The proposed method is validated through comprehensive simulation and demonstrated on real hyperspectral line-scans.

## 1. Introduction

**Figure 1:**
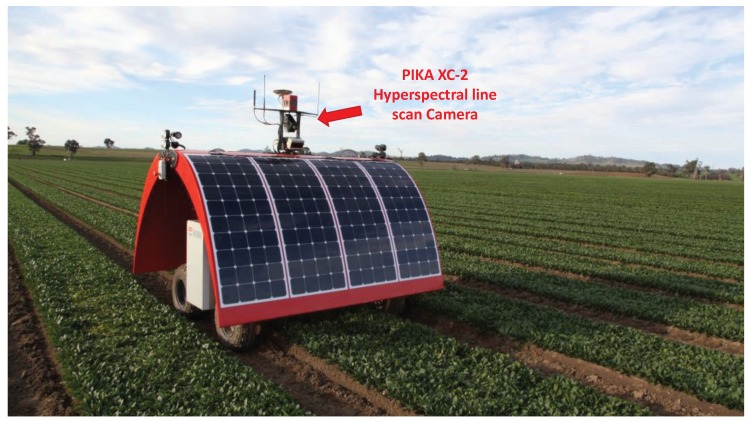
The Ladybird robot developed by Australian Centre for Field Robotics (ACFR) at The University of Sydney.

Hyperspectral line-scan cameras have been widely used by agricultural robots, e.g., the Ladybird robot as shown in Figure 1, for various applications such as fruit detection [1], weed detection [2,3,4], nutrient status estimation [5], pest surveillance [6], discolouration detection [7], damage detection [8], and yield estimation [9]. Hyperspectral line-scan cameras provide both high spatial and spectral resolution and high sample rate images.

Producing hyperspectral images of a scene is a three-dimensional problem. A hyperspectral image provides much more resolution along the spectral dimension compared to panchromatic sensors [10]. Like conventional imaging systems, two dimensions are required to record spatial information. The third dimension is used to record layers of spectral information. Optical systems work by gathering light through a series of lenses and projecting the light onto a two dimensional imaging plane. Capturing the extra dimension of spectral information raises a design challenge. Various technologies have been developed to manage the trade-off between spatial and spectral resolution. Area-scan and line-scan cameras represent different approaches to manage this trade-off. More details about various hyperspectral sensors can be found in [11].

In area-scan cameras, the system iterates through a bank of filters capturing an image for each filter. Each image has two spatial dimensions and represents the reflectance at a particular wavelength. These systems require the scene to remain static as images are captured at each wavelength. They also require complex mechanisms to manage the filter changes. Given that these systems capture two-dimensional data in layers, conventional calibration procedures can be applied to the data. A versatile two-step calibration method is proposed by Tsai to calibrate an area-scan camera by a planar or 3D metric calibration target [12]. A flexible calibration method for an area-scan camera using a printed planar metric calibration target is proposed by Zhang in [13]. Given the ease of use and accuracy of Zhang’s method, it has become a popular method for calibrating desktop vision systems.

In line-scan cameras, one dimension of the imaging plane is dedicated to resolving spatial information and the other is dedicated to resolving spectral information. This is achieved by projecting light from the scene through a slit. This narrow band of light, representing one spatial dimension, is passed through a diffraction grating before being cast onto the imaging plane. These systems are a robust choice for field operations as they are purely optical systems. Whilst this technology can capture line-scans with a high spectral and spatial resolution, the single-spatial dimension data is not immediately recognisable or easy to analyse. In order to generate hyperspectral data products with two spatial dimensions, many line-scan images must be stitched together.

Originally, line scan cameras have been applied only on more controlled and strict conditions like laboratories and satellites [14]. Recently, robotic platforms (e.g., the Ladybird in Figure 1) have been developed and required calibration. Calibration techniques designed to work on area-scan images do not immediately translate to line-scan images. Since only one spatial dimension is recorded in line-scan cameras, the contrast of features within the scene only appears in one direction of the captured image. This makes it difficult to determine the location of feature points on a 2-D calibration target. In turn, estimating the location of the imaging-plane relative to the calibration target becomes difficult to obtain [15].

The rest of the paper is organised as follows. A review of related work is provided in Section 2. The proposed calibration method is described in Section 3. Results from validating the method both in simulation and on real data are presented in Section 4. The performance of the method is discussed in Section 5. Finally, Section 6 presents the conclusions and future work.

## 2. Related Work

Compared to area-scan cameras, relatively few methods for calibrating the intrinsic parameters of line-scan cameras exist. Despite the challenges encountered during calibration, there are several works which propose solutions to this problem.

Horaud et al. [16] proposed a method for calibrating the parameters of a line-scan camera using the line image of a purposefully designed 2D calibration target. The target consists of four co-planar straight lines with three of them parallel and the fourth intercepting the other three with an arbitrary angle. To observe a sufficient number of feature points on the calibration target, a linear stage is used to move the calibration target in two orthogonal directions. The linear stage movements are executed using known increments to control the homography between views. The location of the feature points, in the calibration target frame, are estimated using the cross-ratio. The cross-ratio is constructed by taking the ratio between the product of pair-wise distances of four collinear points. This ratio has the useful property that it is invariant under any projective transform [17].

Luna et al. in [18] extends [16] by utilizing two identical calibration targets. The target consists of two parallel planes, each of which has vertical lines and slanted lines interconnected in a zig-zag configuration. The two targets are parallel with a known offset so that the target is repeated at two different elevations. Since there are sufficient independent feature points, calibration can be done using only one image of the calibration target. This method requires a quasi-coplanarity setup between the sensor and the calibration target.

Li et al. [19] further improved the concept. In their method, the calibration target comprises two orthogonal planes. Similar to [18], two groups of parallel feature lines are drawn on each plane. Using this calibration target, the constraint of a quasi-coplanarity setup between the sensor and the calibration target is removed. As in [19], a single image of the calibration target is sufficient for calibration.

Although these methods are able to calibrate the intrinsic parameters of a line-scan camera model, they all hold the assumption that the lens distortion is small enough to be ignored, or constant within each repeated parallel line. Yao et al. [15] relaxed this assumption and presented a method for calibrating a line-scan camera including the radial distortion of the camera lens. A planar target consisting of repeated vertical and slanted lines is used. A virtual 2D calibration framework is constructed from the 1D image data. Feature point reconstruction is used to transform the 1D camera calibration problem into a 2-D space. In this space, it is possible to use a 2D camera model, with constraints unique to 1D geometry. To estimate the model parameters, two or more views of the calibration target are required.

Methods for calibrating a line-scan camera using a typical chessboard target have also been proposed [20,21]. In these methods, a linear stage is used to scan the calibration target and generate a 2D image. This allows conventional calibration techniques to be applied to the 2D image such as detecting the corner of the chessboard squares. While these methods can provide high precision, they require the scanning rate of the camera to be synchronized with the speed of the stage. Behmann et al. [22] proposed a camera model with a non-linear part that is able to deal with a varying scanning speed during the measurement and also deal with nonlinear distortion. In close range photogrammetry, the equipment required to setup linear stages for calibration are not affordable or convenient to deploy for many low-cost line-scan camera applications [19]. Methods for calibrating a line-scan camera with the help of an auxiliary camera are presented in [23,24].

In this paper, we present an improved cross-ratio invariant-based intrinsic calibration method that is designed to estimate the intrinsic parameters of a hyperspectral line-scan camera including the principal point, focal length, and radial distortion of the lens. This is done by taking multiple images of the calibration target presented in [19] at arbitrary angles. Joint optimization is used to find optimal values for the intrinsic parameters using all images. Compared to previous methods using similar calibration targets, our method is able to estimate radial distortion from multiple view angles without requiring external hardware such as linear stages.

## 3. Calibration Method

**Figure 2:**
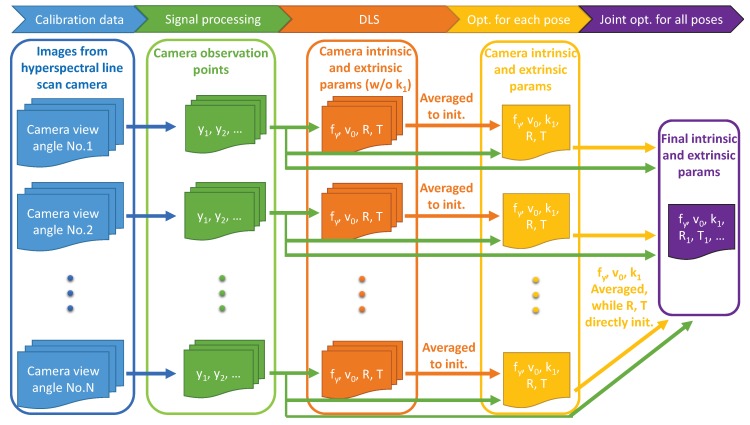
Structure of the proposed method. In the first step, multiple images of the calibration target are gathered at different camera view angles. A signal processing step (Section 3.3) is used to locate calibration feature points in each hyperspectral image. In the next step (Section 3.4), the DLS is used on each set of calibration feature points to estimate the intrinsic and extrinsic parameters of the line-scan camera, excluding radial distortion. At each camera view angle, the DLS estimates are averaged and used as the starting location for a nonlinear least squares optimization of the camera intrinsic and extrinsic parameters including radial distortion (Section 3.5). This is done by minimizing the re-projection errors of the calibration feature points at each camera view angle. A final nonlinear least squares optimization is performed to minimize the re-projection errors of all calibration feature points jointly (Section 3.6). The joint optimisation is initialised using the average intrinsic parameters from all view angles and all extrinsic parameters from the previous step.

This section presents our method for calibrating the intrinsic parameters of a hyperspectral line-scan camera. Section 3.1 and Section 3.2 lay the foundations of the calibration method by introducing the line-scan camera model and details of the calibration target, respectively. The steps of the proposed calibration method are summarised in Figure 2. In the first step, described in Section 3.3, signal processing is used to gather calibration feature points for determining the camera intrinsic parameters. Next, as described in Section 3.4, the calibration feature points are used in the DLS to estimate the camera extrinsic and intrinsic parameters excluding radial distortion. In the next step, described in Section 3.5, nonlinear optimization is used to optimize all camera intrinsic parameters including radial distortion using all calibration data from each camera view angle. Finally, as described in Section 3.6, a joint nonlinear optimization step is performed to optimize intrinsic parameters using all calibration data from all camera view angles.

### 3.1. The Camera Model for a Line-Scan Camera

In the proposed method, the classic pin hole camera model is employed to represent the relationship between a point in the world frame and its correspondence in the camera image. Using a pin hole camera model without considering lens distortion, the 3D projection model of a 2D frame camera is formulated as follows [15]:

(1) $$\begin{array}{c} s\left[\begin{array}{c} u\\ v\\ 1 \end{array}\right]=M\left[\begin{array}{cc} R & t \end{array}\right]\left[\begin{array}{c} X\\ Y\\ Z\\ 1 \end{array}\right] \end{array}$$

where ${\left[\begin{array}{ccc} X & Y & Z \end{array}\right]}^{T}$ are the coordinates of a 3D point in the world frame, ${\left[\begin{array}{cc} u & v \end{array}\right]}^{T}$ is the corresponding pixel coordinates in image, *s* is an arbitrary scale factor, $\left[\begin{array}{cc} R & t \end{array}\right]$ is the extrinsic matrix, and M is the intrinsic matrix of the camera. In the camera extrinsic matrix, R is the rotation matrix and t is the translation matrix specifying the relative pose between the world coordinates frame and the camera coordinates frame.

The intrinsic matrix M is defined by

(2) $$\begin{array}{c} M=\left[\begin{array}{ccc} f_{x} & c & u_{0}\\ 0 & f_{y} & v_{0}\\ 0 & 0 & 1 \end{array}\right] \end{array}$$

where $f_{x}$ and $f_{y}$ are horizontal and vertical focal lengths in pixels, *c* is the skewness of the axes in the image frame, and $u_{0}$ and $v_{0}$ are the coordinates of the principal point on the image.

Projection of a line-scan camera can be treated as a special case of frame camera projection. For the 1D line-scan camera, one can assume that the camera optical center locates on the line-scan camera’s sensor array, i.e., $u_{0}=0$, and the skewness of the axes has no effect, i.e., c=0 [15]. Therefore, Equations (1) and (2) for a line-scan camera can be simplified to

(3) $$\begin{array}{c} s\left[\begin{array}{c} 0\\ v\\ 1 \end{array}\right]=\left[\begin{array}{ccc} f_{x} & 0 & 0\\ 0 & f_{y} & v_{0}\\ 0 & 0 & 1 \end{array}\right]\left[\begin{array}{cc} R & t \end{array}\right]\left[\begin{array}{c} X\\ Y\\ Z\\ 1 \end{array}\right]. \end{array}$$

Let $r_{ij}$ be the *i*th row and the *j*th column of the rotation matrix R, and let $t_{i}$ be the *i*th element of the translation matrix t. Equation (3) can be further simplified as

(4) $$\left\{\begin{array}{c} 0=f_{x}\frac{r_{11}X+r_{12}Y+r_{13}Z+t_{1}}{r_{31}X+r_{32}Y+r_{33}Z+t_{3}}\\ v=f_{y}\frac{r_{21}X+r_{22}Y+r_{23}Z+t_{2}}{r_{31}X+r_{32}Y+r_{33}Z+t_{3}}+v_{0} \end{array}\right..$$

When the distortion of camera lens is taken into account, we consider the first-order radial distortion coefficient. Since the image captured by a line-scan camera only contains one spatial dimension, the tangential distortion of the lens does not affect the image [15]. Although the radial distortion can spread over more than 20 orders of magnitude, it is dominated by the first order [12,25]. As a result, we can add the effect of the first-order radial distortion of the lens to Equation (4) and obtain the following formulation:

(5) $$\left\{\begin{array}{c} 0=f_{x}\frac{r_{11}X+r_{12}Y+r_{13}Z+t_{1}}{r_{31}X+r_{32}Y+r_{33}Z+t_{3}}\\ v=f_{y}\left(\frac{r_{21}X+r_{22}Y+r_{23}Z+t_{2}}{r_{31}X+r_{32}Y+r_{33}Z+t_{3}}\left(1+k_{1}{\left(\frac{r_{21}X+r_{22}Y+r_{23}Z+t_{2}}{r_{31}X+r_{32}Y+r_{33}Z+t_{3}}\right)}^{2}\right)\right)+v_{0} \end{array}\right.$$

where $k_{1}$ is the first-order radial distortion coefficient. In Equation (5), the first equation is not affected by the radial distortion, as there is only one pixel along this direction. We use Equation (4) for the DLS and Equation (5) for the nonlinear optimization, as detailed in later sections (Section 3.4, Section 3.5 and Section 3.6).

### 3.2. Calibration Target

We adopt the same calibration target as designed by Li et al. in [19]. The benefit of such a target over other calibration targets reported in the literature is that it is easy to manufacture in a small workshop. Only one image of the calibration target is required to estimate the camera’s extrinsic and intrinsic parameters, excluding the radial distortion coefficient, using the DLS described in Section 3.4.

**Figure 3:**
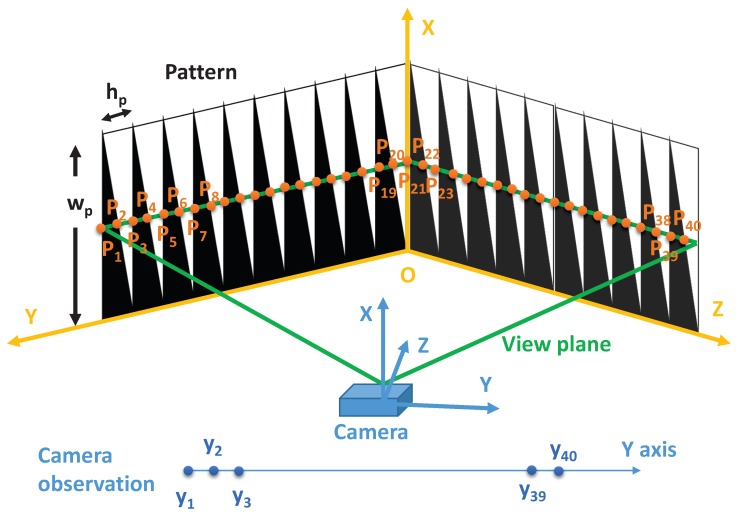
Calibration target used in the proposed method. $w_{p}$ and $h_{p}$ are the width and height of the triangles. The triangles are repeated using a constant offset of $h_{p}$. The coordinate frame of the calibration target is denoted in orange and the coordinate frame of the camera is denoted in blue. The view plane of the line-scan camera is shown in green and intersects with the black triangles at points $P_{i}\left(i=1,\cdots ,40\right)$.

The calibration target is shown in Figure 3. As can be seen from the figure, the target consists of two orthogonal planes. Each plane is painted with 10 black triangles. The width $w_{p}$ and height $h_{p}$ of the triangles are known, allowing equations for the vertical and diagonal lines in the target to be modelled prior to imaging. The line-scan camera’s view plane (denoted in green) intersects with the black triangles on the points $P_{i}\left(i=1,\cdots ,40\right)$. The projected points $P_{i}\left(i=1,\cdots ,40\right)$ in the line-scan camera image frame are denoted as $y_{i}\left(i=1,\cdots ,40\right)$.

In a practical calibration process, one can only obtain the camera observation $y_{i}\left(i=1,\cdots ,40\right)$. However, when neglecting the effect of lens distortion, 3D points coordinates of $P_{i}\left(i=1,\cdots ,40\right)$ can be inferred using the cross-ratio invariance property [19].

To obtain the coordinates at $P_{2}$, let us firstly define the cross ratio of the points $P_{1}$, $P_{2}$, $P_{3}$, $P_{5}$ as $\eta_{P_{1},P_{2},P_{3},P_{5}}$ such that

(6) $$\eta_{P_{1},P_{2},P_{3},P_{5}}=\frac{P_{1}P_{3}}{P_{2}P_{3}}:\frac{P_{1}P_{5}}{P_{2}P_{5}}=\frac{P_{1}P_{2}+P_{2}P_{3}}{P_{2}P_{3}}:\frac{P_{1}P_{2}+P_{2}P_{3}+P_{3}P_{4}+P_{4}P_{5}}{P_{2}P_{3}+P_{3}P_{4}+P_{4}P_{5}}.$$

Due to the parallelism of the vertical lines on the target, the following relationship holds:

(7) $$P_{3}P_{4}+P_{4}P_{5}=P_{1}P_{2}+P_{2}P_{3}.$$

Therefore, Equation (6) can be simplified as

(8) $$\eta_{P_{1},P_{2},P_{3},P_{5}}=\frac{P_{1}P_{2}+P_{2}P_{3}}{P_{2}P_{3}}:\frac{2(P_{1}P_{2}+P_{2}P_{3})}{P_{1}P_{2}+2P_{2}P_{3}}.$$

From the projective invariance property of the cross ratio, we can also express $\eta_{P_{1},P_{2},P_{3},P_{5}}$ as [19]:

(9) $$\eta_{P_{1},P_{2},P_{3},P_{5}}=\frac{y_{3}-y_{1}}{y_{3}-y_{2}}:\frac{y_{5}-y_{1}}{y_{5}-y_{2}}.$$

Therefore, using Equations (8) and (9),

(10) $$\frac{P_{2}P_{3}}{P_{1}P_{3}}=\frac{\left(y_{3}-y_{2}\right)\left(y_{5}-y_{1}\right)}{2\left(y_{3}-y_{1}\right)\left(y_{5}-y_{2}\right)-\left(y_{3}-y_{2}\right)\left(y_{5}-y_{1}\right)}.$$

Finally, using parallelism again, the coordinates of $P_{2}$ in the target coordinate frame can be derived as

(11) $$\left\{\begin{array}{c} X_{2}=w_{p}\frac{P_{2}P_{3}}{P_{1}P_{3}}\\ Y_{2}=h_{p}\left(9+\frac{P_{2}P_{3}}{P_{1}P_{3}}\right)\\ Z_{2}=0 \end{array}\right.$$

where $\frac{P_{2}P_{3}}{P_{1}P_{3}}$ is obtained from Equation (10).

Similarly, the coordinates of points $P_{i}\left(i=4,6,\cdots ,16\right)$ and $P_{i}\left(i=22,24,\cdots ,36\right)$ can be obtained as follows [19]:

(12) $$\left\{\begin{array}{c} X_{i}=w_{p}\frac{P_{i}P_{i+1}}{P_{i-1}P_{i+1}}\\ Y_{i}=h_{p}\left(\frac{20-i}{2}+\frac{P_{i}P_{i+1}}{P_{i-1}P_{i+1}}\right)\\ Z_{i}=0 \end{array}\right.,\;i=2,4,\cdots ,16$$

(13) $$\left\{\begin{array}{c} X_{i}=w_{p}\frac{P_{i}P_{i+1}}{P_{i-1}P_{i+1}}\\ Y_{i}=0\\ Z_{i}=h_{p}\left(\frac{i-20}{2}-\frac{P_{i}P_{i+1}}{P_{i-1}P_{i+1}}\right) \end{array}\right.,\;i=22,24,\cdots ,36$$

where

(14) $$\frac{P_{i}P_{i+1}}{P_{i-1}P_{i+1}}=\frac{\left(y_{i+1}-y_{i}\right)\left(y_{i+3}-y_{i-1}\right)}{2\left(y_{i+1}-y_{i-1}\right)\left(y_{i+3}-y_{i}\right)-\left(y_{i+1}-y_{i}\right)\left(y_{i+3}-y_{i-1}\right)},i=2,4,\cdots ,16;22,24,\cdots ,36.$$

Ideally, $P_{i}\left(i=2,4,\cdots ,16;22,24,\cdots ,36\right)$ should be perfectly aligned on the view plane. However, due to observation noise on $y_{i}\left(i=2,4\cdots ,16;22,24,\cdots ,36\right)$, $P_{i}\left(i=2,4\cdots ,16;22,24,\cdots ,36\right)$ are recorded with minor offsets from the view plane. The equation of view plane can be defined in the coordinates of target frame as AX+BY+CZ+D=0. The coefficients *A*,*B*,*C*, and *D* can be refined by solving the following homogeneous linear equations using singular value decomposition [19]:

(15) $$\begin{array}{c} \left[\begin{array}{cccc} X_{i} & Y_{i} & Z_{i} & 1\\ \vdots & \vdots & \vdots & \vdots \\ X_{N} & Y_{N} & Z_{N} & 1 \end{array}\right]\left[\begin{array}{c} A\\ B\\ C\\ D \end{array}\right]=0. \end{array}$$

After solving Equation (15) to estimate the equation of the view plane, the location of the points $P_{i}\left(i=1,\cdots ,40\right)$ can be estimated by solving for the intersection between the 3D plane and lines. This is given by the following equations:

(16) $$\;\left\{\begin{array}{c} X_{i}=-\frac{BY_{i}+D}{A}\\ Y_{i}=h_{p}\left(\frac{21-i}{2}\right)\\ Z_{i}=0 \end{array}\right.,\;i=1,3,\cdots ,19$$

(17) $$\;\left\{\begin{array}{c} X_{i}=-\frac{B\left(\frac{20-i}{2}\right)h_{p}+D}{A+\frac{Bh_{p}}{w_{p}}}\\ Y_{i}=-\frac{AX_{i}+D}{B}\\ Z_{i}=0 \end{array}\right.,\;i=2,4,\cdots ,20$$

(18) $$\;\left\{\begin{array}{c} X_{i}=-\frac{CZ_{i}+D}{A}\\ Y_{i}=0\\ Z_{i}=\frac{h_{p}\left(i-21\right)}{2} \end{array}\right.,\;i=21,23,\cdots ,39$$

(19) $$\;\left\{\begin{array}{c} X_{i}=-\frac{C\left(\frac{i-20}{2}\right)h_{p}+D}{A-\frac{Ch_{p}}{w_{p}}}\\ Y_{i}=0\\ Z_{i}=-\frac{AX_{i}+D}{C} \end{array}\right.,\;i=22,24,\cdots ,40.$$

More details about the derivation of the above equations can be found in [19].

### 3.3. Signal Processing

Before calibration, described in later sections, the observations $y_{i}\left(i=1,\cdots ,40\right)$ of points $P_{i}\left(i=1,\cdots ,40\right)$ must be extracted from the raw hyperspectral imagery.

**Figure 4:**
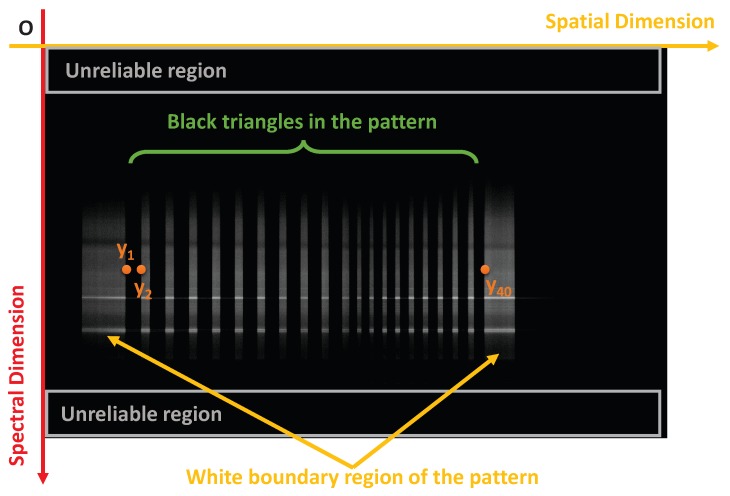
An image captured by a hyperspectral line-scan camera facing the calibration target shown in Figure 3. The camera gathers data across the view plane. In the 2D image returned by the camera, the *x*-axis records spatial information across the view plane and the *y*-axis records spectral information.

A hyperspectral line-scan image of the calibration target is shown in Figure 4. As can be seen from the figure, the camera provides a 2D image with one axis representing the spatial dimension, and the other representing the spectral dimension. To calibrate the camera, all vertical edges in the image must be located. These edges correspond to the projected intersection points $y_{i}\left(i=1,\cdots ,40\right)$ where the black and white pigment meet along the view plane of the target *i.e.* $P_{i}\left(i=1,\cdots ,40\right)$. Here, we assume that the consequences of smiling and keystone effects on the vertical edge detection is negligible.

Let I(i,j) be the image from the hyperspectral line-scan camera, where indexes *i* and *j* represent indexes along spectral and spatial dimensions, respectively. Pixels close to the top and bottom of the spectral dimension are not considered, as the signal has attenuated to below the noise floor. Specifically, pixels correspond to wavelength less than 420nm or larger than 950nm are ignored. For each line along the spatial dimension, the gradient is calculated with respect to adjacent pixels and summed to calculate a gradient score value g(i,j) where

(20) g(i,j)=∥I(i,j)−I(i,j−1)∥+∥I(i,j)−I(i,j+1)∥.

The gradient score, g(i,j), is summed along the spectral dimension to obtain the spatial gradient score value G(j),

(21) $$G\left(j\right)=\sum_{i=J_{l}}^{J_{u}}g\left(i,j\right)$$

where $J_{l}$ and $J_{u}$ denote the lower and upper indexes of the reliable region of the image, respectively.

**Figure 5:**
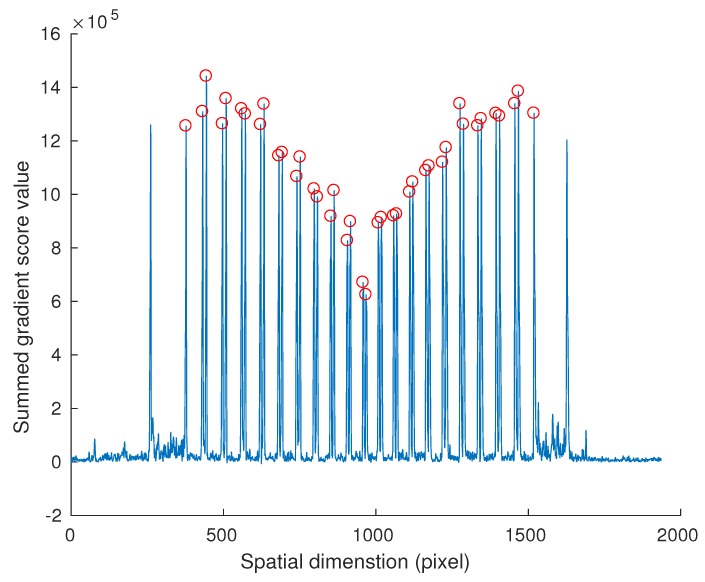
Estimation of camera observations points $y_{i}\left(i=1,\cdots ,40\right)$ from a hyperspectral line-scan camera image. The blue line is the up-sampled and cubic spline interpolated gradient score value G(j). The red circles are estimated camera observation point $y_{i}\left(i=1,\cdots ,40\right)$. The camera observation points are obtained by extracting the 42 highest peaks and excluding the first and last peaks along the spatial dimension. The first and last peaks correspond to the edges of the calibration target.

In order to find $y_{i}\left(i=1,\cdots ,40\right)$ with subpixel accuracy, G(j) are up-sampled along the spatial dimension and fitted using cubic spline interpolation [26] as shown in Figure 5. As shown in the figure, there are 42 distinctive peaks representing the 40 points that constitute $y_{i}\left(i=1,\cdots ,40\right)$. The two extra points represent the edge of the calibration target. Therefore, we extract the 42 highest peaks and exclude the first and last peaks along the spatial dimension. The obtained peaks are scaled back to the original pixel coordinate scale to obtain $y_{i}\left(i=1,\cdots ,40\right)$.

### 3.4. Direct Linear Solution

When neglecting the lens distortion of the line-scan camera, extrinsic and intrinsic camera parameters can be obtained using the DLS on one image of the calibration target [19].

Using the DLS, the extrinsic and intrinsic parameters in Equation (4) can be formulated as follows:

(22) $$\left\{\begin{array}{c} r_{11}=-\frac{DA}{∥D∥\sqrt{A^{2}+B^{2}+C^{2}}}\\ r_{12}=-\frac{DB}{∥D∥\sqrt{A^{2}+B^{2}+C^{2}}}\\ r_{13}=-\frac{DC}{∥D∥\sqrt{A^{2}+B^{2}+C^{2}}}\\ t_{1}=-\frac{D^{2}}{∥D∥\sqrt{A^{2}+B^{2}+C^{2}}} \end{array}\right.$$

(23) $$\left\{\begin{array}{c} r_{21}=\frac{s(l_{4}r_{13}-l_{5}r_{12})}{r_{11}}\\ r_{22}=sl_{5}\\ r_{23}=-sl_{4} \end{array}\right.$$

(24) $$\left[\begin{array}{c} r_{31}\\ r_{32}\\ r_{33} \end{array}\right]=\left[\begin{array}{c} r_{11}\\ r_{12}\\ r_{13} \end{array}\right]\times \left[\begin{array}{c} r_{21}\\ r_{22}\\ r_{23} \end{array}\right]$$

where

(25) $$s=\pm \frac{1}{\sqrt{l_{5}^{2}+l_{4}^{2}+{\left(\frac{l_{4}r_{13}-l_{5}r_{12}}{r_{11}}\right)}^{2}}}.$$

$l_{i}\left(i=1,\cdots ,6\right)$ can be obtained by solving the following homogeneous linear equations using singular value decomposition:

(26) $$\left[\begin{array}{cccccc} Y_{1} & Z_{1} & 1 & -y_{1}Y_{1} & -y_{1}Z_{1} & -y_{1}\\ \vdots & \vdots & \vdots & \vdots & \vdots & \vdots \\ Y_{N} & Z_{N} & 1 & -y_{N}Y_{N} & -y_{N}Z_{N} & -y_{N} \end{array}\right]\left[\begin{array}{c} l_{1}\\ l_{2}\\ l_{3}\\ l_{4}\\ l_{5}\\ l_{6} \end{array}\right]=0.$$

Finally, parameters $f_{y}$,$v_{0}$, $t_{2}$, and $t_{3}$ can be obtained by solving the following equations:

(27) $$\left\{\begin{array}{c} f_{y}r_{33}-v_{0}r_{23}=sl_{1}\\ -f_{y}r_{32}+v_{0}r_{22}=sl_{2}\\ f_{y}r_{11}t_{2}-f_{y}r_{21}t_{1}+v_{0}r_{11}t_{3}-v_{0}r_{31}t_{1}=sl_{3}\\ r_{11}t_{3}-r_{31}t_{1}=sl_{6} \end{array}\right..$$

Equation (25) has two solutions for *s*. The incorrect solution for *s* can be discarded by checking the sign of $t_{3}$. Since the calibration target is guaranteed to be in front of the line-scan camera and $t_{3}$ represents the *Z* axis value of the camera in the target coordinate frame, we can enforce $t_{3}>0$. By ensuring $t_{3}>0$, we can obtain the correct, unique solution. More details on the derivation of the DLS can be found in [19].

### 3.5. Nonlinear Optimization for Each Camera Pose

The results of the DLS has several drawbacks. Firstly, the estimated coordinates of intersection points $P_{i}$ are not the true intersection points of the camera view plane and the patterns on the target. Secondly, the camera observations $y_{i}$ and the camera model do not satisfy the optimal least squares measure [19].

With the DLS extrinsic and intrinsic camera parameters obtained in Section 3.4, the parameters can be updated and refined to include radial distortion of the camera lens using nonlinear optimization. All calibration data from one camera pose is used in this nonlinear optimization step.

In the nonlinear optimization, the following sum of re-projection errors are minimized:

(28) $$e=\sum_{\delta =1}^{\Delta }\sum_{i=1}^{40}\left[{\left({\hat{x}}_{i}\left(R,T,f_{x},k_{1}\right)-0\right)}^{2}+{\left({\hat{y}}_{i}\left(R,T,f_{y},k_{1}\right)-y_{i,\delta }\right)}^{2}\right]$$

where ${\hat{x}}_{i}\left(R,T,f_{x},k_{1}\right)$ and ${\hat{y}}_{i}\left(R,T,f_{y},k_{1}\right)$ are the expected camera observations of intersection points $P_{i}$, Δ is the number of calibration data in each camera view angle, and $y_{i,\delta }$ is the actual camera observation of the intersection points $P_{i}$ in the δth calibration data. ${\hat{x}}_{i}\left(R,T,f_{x},k_{1}\right)$ and ${\hat{y}}_{i}\left(R,T,f_{y},k_{1}\right)$ are functions of the camera extrinsic parameters, including rotation *R*, translation *T*, focal lengths $f_{x}$ and $f_{y}$, and the first-order radial distortion coefficient $k_{1}$ of the camera lens. Note that, in the proposed method, ${\hat{x}}_{i}$ and ${\hat{y}}_{i}$ are not functions of $P_{i}$, as opposed to the conventional approach [19]. This is because, once the rotation *R* and the translation *T* of the camera pose are fixed, the view plane of the line-scan camera is fixed. Therefore, the intersections points $P_{i}$ are simply functions of camera extrinsic parameters *R* and *T*, i.e.,

(29) $$P_{i}=f_{i}\left(R,T\right).$$

As points $P_{i}$ are fully determined by *R* and *T*, inserting them into ${\hat{x}}_{i}$ and ${\hat{y}}_{i}$ makes the optimization over-parametrized in the conventional approach. The function $f_{i}\left(R,T\right)$ can be obtained using 3D plane lines intersection, since the camera view plane is determined by *R* and *T*, and the line equations in the calibration target are known prior. As $P_{i}$ are no longer arguments of ${\hat{x}}_{i}$ and ${\hat{y}}_{i}$, the proposed approach does not need to explicitly update the coordinates of $P_{i}$ at each optimization iteration as in the conventional approach [19]. They are implicitly updated along with the change of *R* and *T*.

Using the defined camera model in Equation (5), the expected camera observation ${\hat{x}}_{i}\left(R,T,f_{x},k_{1}\right)$ and ${\hat{y}}_{i}\left(R,T,f_{y},k_{1}\right)$ in Equation (28) can be formulated as follows:

(30) $$\left\{\begin{array}{c} {\hat{x}}_{i}\left(R,T,f_{x},k_{1}\right)=f_{x}\frac{r_{11}X_{i}+r_{12}Y_{i}+r_{13}Z_{i}+t_{1}}{r_{31}X_{i}+r_{32}Y_{i}+r_{33}Z_{i}+t_{3}}\\ {\hat{y}}_{i}\left(R,T,f_{x},k_{1}\right)=f_{y}\left(\frac{r_{21}X_{i}+r_{22}Y_{i}+r_{23}Z_{i}+t_{2}}{r_{31}X_{i}+r_{32}Y_{i}+r_{33}Z_{i}+t_{3}}\left(1+k_{1}{\left(\frac{r_{21}X_{i}+r_{22}Y_{i}+r_{23}Z_{i}+t_{2}}{r_{31}X_{i}+r_{32}Y_{i}+r_{33}Z_{i}+t_{3}}\right)}^{2}\right)\right)+v_{0} \end{array}\right..$$

Since the line-scan camera only records one spatial dimension, it is not necessary to model $f_{x}$. The parameter is only required to scale the reprojection error ${\hat{x}}_{i}\left(R,T,f_{x},k_{1}\right)-0$. For practical optics, $f_{x}\approx f_{y}$, so Equation (30) can be simplified to

(31) $$\left\{\begin{array}{c} {\hat{x}}_{i}\left(R,T,f_{x},k_{1}\right)=f_{y}\frac{r_{11}X_{i}+r_{12}Y_{i}+r_{13}Z_{i}+t_{1}}{r_{31}X_{i}+r_{32}Y_{i}+r_{33}Z_{i}+t_{3}}\\ {\hat{y}}_{i}\left(R,T,f_{x},k_{1}\right)=f_{y}\left(\frac{r_{21}X_{i}+r_{22}Y_{i}+r_{23}Z_{i}+t_{2}}{r_{31}X_{i}+r_{32}Y_{i}+r_{33}Z_{i}+t_{3}}\left(1+k_{1}{\left(\frac{r_{21}X_{i}+r_{22}Y_{i}+r_{23}Z_{i}+t_{2}}{r_{31}X_{i}+r_{32}Y_{i}+r_{33}Z_{i}+t_{3}}\right)}^{2}\right)\right)+v_{0} \end{array}\right..$$

Note that $X_{i}$, $Y_{i}$, and $Z_{i}$ are the *X*, *Y*, and *Z* coordinates of points $P_{i}$ that can be obtained from Equation (29) using *R* and *T*. Furthermore, in the proposed method, both ${\hat{x}}_{i}$ and ${\hat{y}}_{i}$ in Equation (31) are properly scaled to obtain the re-projection errors in pixels. In the conventional method only, ${\hat{y}}_{i}$ is scaled to represent the re-projection error in pixels. This means the overall accumulated least squares error has different weights in the *X*- and *Y*-directions, which in our opinion is sub-optimal.

Equations (28) and (31) form a classic least squares optimization problem minimizing the camera re-projection error where the Levenberg–Marquardt algorithm [27] can be used to solve the problem. The parameters to be optimized are the camera intrinsic parameters $f_{y}$, $v_{0}$, and $k_{1}$ and the extrinsic parameters *R* and *T*. As mentioned before, the intersection points $P_{i}$ do not need to be updated at each iteration step, since they are implicitly updated due to the change in *R* and *T*.

### 3.6. Joint Nonlinear Optimization for Multi Camera Poses

The nonlinear optimization in Section 3.5 only uses observations from a single camera view angle. This is problematic as the parameters have different sensitivities to different view angles. To minimize this effect, we use calibration data from different camera view angles and jointly optimize all extrinsic and intrinsic parameters. The error of this joint nonlinear optimization can be defined by extending Equation (28) such that

(32) $$e_{joint}=\sum_{\zeta =1}^{Z}\sum_{\delta =1}^{\Delta }\sum_{i=1}^{40}\left[{\left({\hat{x}}_{\zeta ,i}\left(R_{\zeta },T_{\zeta },f_{x},k_{1}\right)-0\right)}^{2}+{\left({\hat{y}}_{\zeta ,i}\left(R_{\zeta },T_{\zeta },f_{y},k_{1}\right)-y_{\zeta ,i,\delta }\right)}^{2}\right]$$

where the subscript ζ represents a different camera view angle. The terms ${\hat{x}}_{\zeta ,i}\left(R_{\zeta },T_{\zeta },f_{x},k_{1}\right)$ and ${\hat{y}}_{\zeta ,i}\left(R_{\zeta },T_{\zeta },f_{y},k_{1}\right)$ can be computed using Equations (31) and (29), and different $R_{\zeta }$ and $T_{\zeta }$ for each camera view angle ζ=1,⋯,Z.

Note that results of this joint optimization step cannot be achieved by simply averaging the optimization results from Section 3.5 over all camera view angles. Since $f_{y}$ and $k_{1}$ are independent of the camera view angle, they should be modelled as a constant across all observations. The proposed method is able to model correctly the camera view angle dependent and independent terms while making full use of the observation data. This results in a solution that is both accurate and robust to observation.

## 4. Validation

In this section, the proposed calibration method is validated through simulation in Section 4.1 and a calibration using real hyperspectral line-scan data in Section 4.2.

### 4.1. Simulation

In real-world calibration, the task is to estimate the true value of a set of parameters through indirect observation and optimisation. As a result, it is difficult to assess how close the estimated parameters are to their true values. Additionally, real-world calibration tasks are labour-intensive, making it time-consuming to vary parameters during data collection. To analyse the proposed calibration method in a highly repeatable environment with access to ground truth measurements, we developed a simulation.

**Figure 6:**
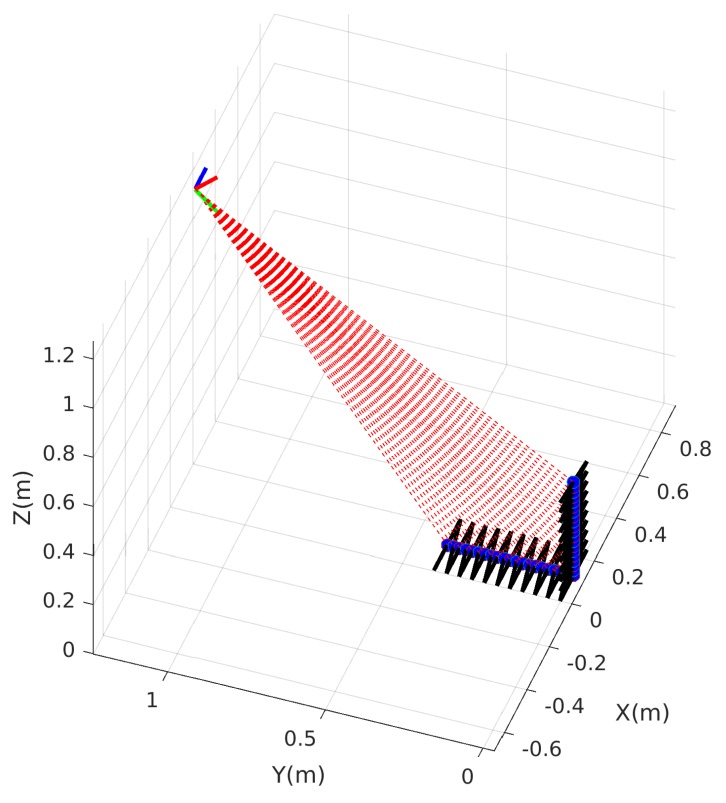
Simulation of a line-scan camera facing a calibration target. The line-scan camera is represented by the blue, red, and green axes, which represent *X*, *Y*, and *Z*-axes of the line-scan camera, respectively. The camera view plane is represented by red dash lines. Contours of triangles in the calibration target are represented by black lines. The intersection points of the camera view plane and contours of black triangles are denoted by blue dots. The *X*, *Y*, and *Z*-axes of the figure represent the coordinate frame of the target.

The simulation models a line-scan camera facing the calibration target shown in Figure 3. An illustration of the simulation is shown in Figure 6. The parameters of the simulation are summarized in Table 1 and similar to those use in [19]. In the simulation, 15 camera view angles and 100 images per view angle are generated to compare the performance of the conventional method [19], the proposed method without joint optimization (Section 3.5) and the proposed method with joint optimization using multiple camera view angles (Section 3.6). In practice, calibration data from more number of and more widely distributed view angles is more helpful in reducing the estimation errors. Empirically, we found calibration data from more than 10 widely distributed view angles can yield satisfying results.

To obtain results using the conventional method, the estimates from all 100 images in each camera view angle are averaged to reduce the error. As there are 15 camera view angles, 15 estimates are obtained. The conventional method was re-implemented and follows the DLS and nonlinear optimization with the intersection points $P_{i}$ update steps documented in the paper. However, we change the nonlinear optimization step to include radial distortion $k_{1}$ so that a fair comparison can be made with our proposed method. In our proposed method *without* joint optimization, the camera parameters are obtained by optimizing 100 images in each camera view angle. Again, 15 estimates are obtained. In the proposed method *with* joint optimization, 100 images from 14 camera view angles are jointly optimized. Using this method, 15 different estimates from 15 different combinations of camera view angles can be obtained.

Three simulated experiments were performed. Section 4.1.1 examines the affect of observation noise on the estimated extrinsic and intrinsic parameters. Section 4.1.2 models the affect of a systematic bias in the observation on the estimated intrinsic parameters. Finally, Section 4.1.3 shows the effect of poor tolerances in the calibration target on the estimated intrinsic parameters. In all of the following figures in this section, plots in blue are the results of the conventional method, red plots are the results of the proposed method without joint optimization, and green plots are the results of the proposed method with joint optimization. In the following box and whisker plots, the bottom and top edges of each box indicate the 25% and 75% percentiles, respectively, and the central mark indicates the median. The whiskers extend to the most extreme data points not considered outliers. Outliers are plotted individually using the ’+’ symbol.

#### 4.1.1. Noisy Camera Observations

The effect of observation noise on $y_{i}$ is tested by adding increasing levels of Gaussian noise, ranging from 0.2 to 2 pixel to the observations.

**Figure 7:**
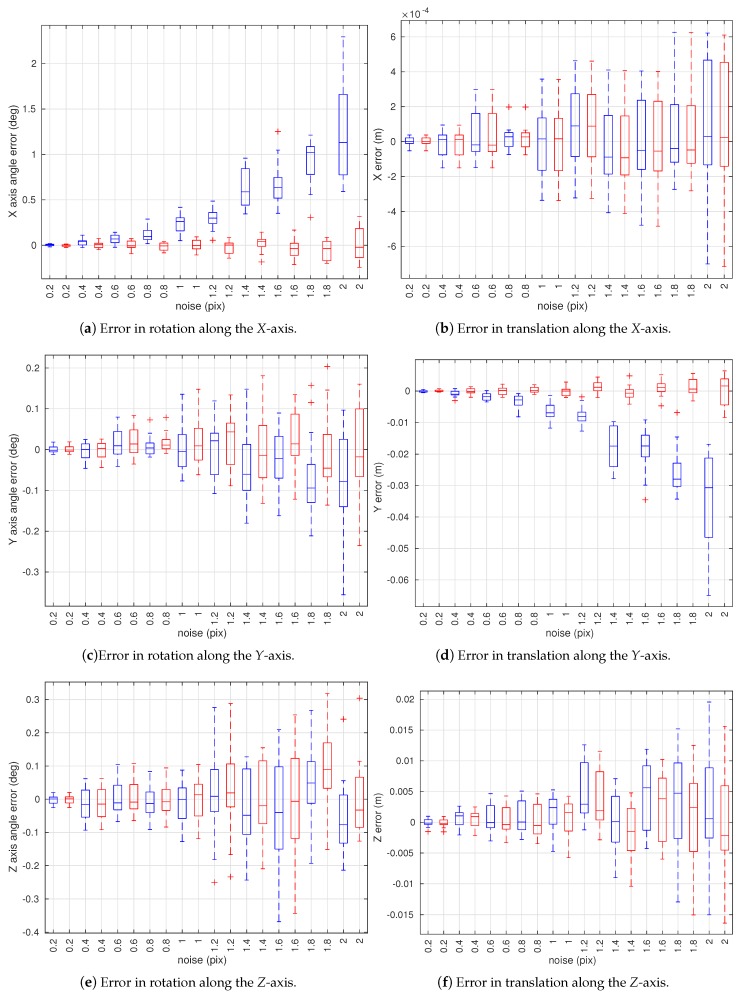
Box plots of estimation errors in camera extrinsic parameters w.r.t. camera observation noise. The box plots in blue represent estimation results using the conventional method [19] and red ones are of the proposed method without joint optimization.

Figure 7 shows the statistics of the error in the extrinsic parameter estimates. Extrinsic calibration results of the proposed method with joint optimization are not shown since not all extrinsic parameters are estimated at each combination of camera view angles. The data show that the proposed method without joint optimization always produces estimates that are similar to, or better than, the conventional method. In particular, the proposed method without joint optimization substantially outperforms the conventional method in rotation along the *X*-axis (Figure 7a) and translation along the *Y*-axis (Figure 7d). In the conventional method, the error in these values grows as the magnitude of the observation noise increases. In our proposed method, the error remains relatively constant with a tighter spread as the noise level increases.

**Figure 8:**
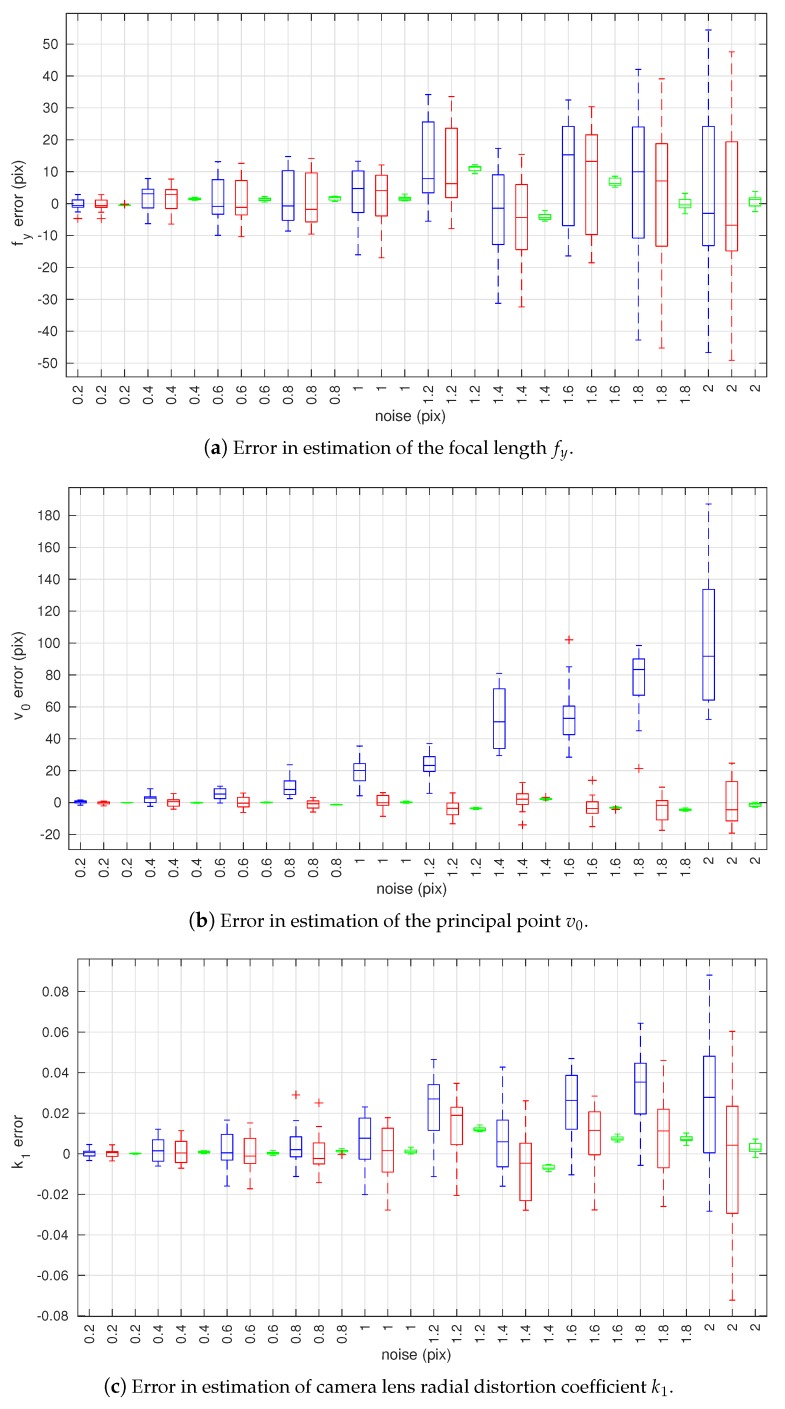
Box plots of estimation errors in camera intrinsic parameters w.r.t. camera observation noise. The box plots in blue represent estimation results using the conventional method [19], red plots are of the proposed method without joint optimization, and green plots are of the proposed method with joint optimization.

**Figure 9:**
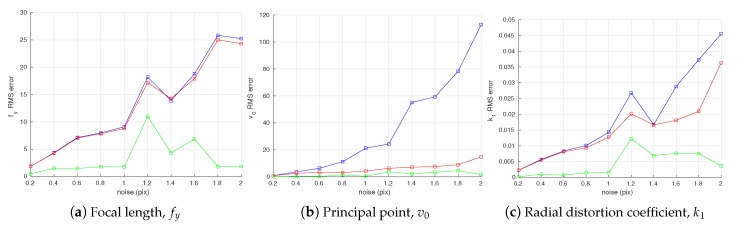
Root-mean-square error (RMSE) in camera intrinsic parameters w.r.t. camera observation noise. The blue, red, and green plots represent RMSE of the conventional method [19], the proposed method without joint optimization and the proposed method with joint optimization, respectively.

The statistics of the error in the intrinsic parameter estimates are shown in Figure 8. All three methods are able to achieve a similar accuracy when estimating the focal length $f_{y}$ (Figure 8a) and the radial distortion coefficient $k_{1}$ (Figure 8c). For both parameters, the joint optimization method holds a small advantage. Both the proposed optimization methods are capable of producing more accurate estimates of principal point $v_{0}$ (Figure 8b). These trends are confirmed by the RMSE of the camera intrinsic parameter estimates as shown in Figure 9.

A clear difference in the results is that the joint optimization method is able to produce estimates that yield a lower spread of error values (Figure 8). This advantage is due to proper handling of the extrinsic parameters as variable over different view angles and the intrinsic parameters as fixed across all view angles. By optimising over all data, the method is both robust to noise and less prone to view angle dependent biases.

#### 4.1.2. Noisy Camera Observations with Bias

The effect of systematic error during the signal processing detailed in Section 3.3 is tested by adding increasing offsets to the camera observations $y_{i}$. In each simulation, the spread of the noise is held at a standard deviation of one pixel while the mean offset is increased.

**Figure 10:**
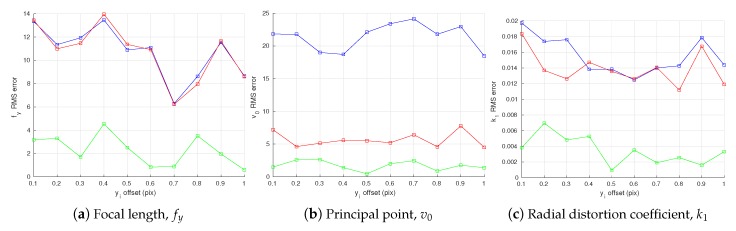
Root-mean-square error (RMSE) in camera intrinsic parameters w.r.t. systematic errors in estimation of camera observation $y_{i}$. The blue, red, and green plots represent RMSE of the conventional method [19], the proposed method without joint optimization and the proposed method with joint optimization, respectively.

The results are shown in Figure 10. It can be seen from the figure that the proposed method with joint optimization outperforms the other two in estimation of all three intrinsic parameters. The accuracy of the conventional method and the proposed method without joint optimization are similar in estimating the focal length $f_{y}$ and the radial distortion $k_{1}$ parameters. Both proposed methods clearly yield better accuracy in estimation of the principal point $v_{0}$. In Figure 10a,c, some RMS estimation errors of larger offsets are smaller than those of smaller offsets. We think that this is due to the randomness of Gaussian noise added to the observations in simulation.

#### 4.1.3. Calibration Target Error

Finally, the calibration methods are subject to error in the calibration board. Although the calibration target has relatively simple geometry, it can be difficult to manufacture a large, light-weight target with perfectly perpendicular planes. Given the angle between the two planes is likely to be the largest source of error during construction, this error is simulated. This is done by adding errors, ranging from 0 to 0.9 degrees, to the angle between two planes. In addition, Gaussian noise with one pixel standard deviation is added to the camera observation $y_{i}$.

**Figure 11:**
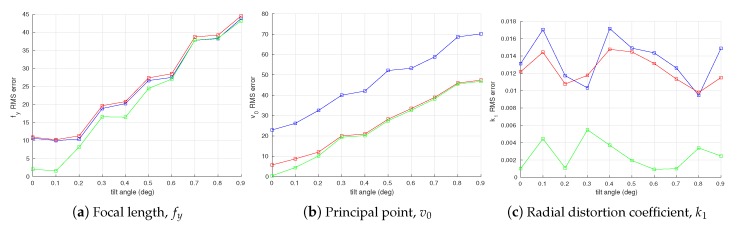
Root-mean-square error (RMSE) in camera intrinsic parameters w.r.t. error in the angle between two planes of the calibration target. The blue, red, and green plots represent RMSE of the conventional method [19], the proposed method without joint optimization, and the proposed method with joint optimization, respectively.

Figure 11 shows the effect that the error in the angle between the calibration target planes has on the RMSE of the intrinsic parameters. We can see from Figure 11a that all three methods yield similar accuracies in estimating the focal length $f_{y}$, while the proposed method with joint optimization performs slightly better when the error in the angle is small. For the principal point $v_{0}$ in Figure 11b, both proposed methods clearly outperform the conventional method, with the proposed method with joint optimization slightly better than the one without. Finally, for estimation of radial distortion $k_{1}$, the proposed method without joint optimization is slighter better than the conventional method, while the joint optimization clearly outperforms the other two.

### 4.2. Experimental Results

**Figure 12:**
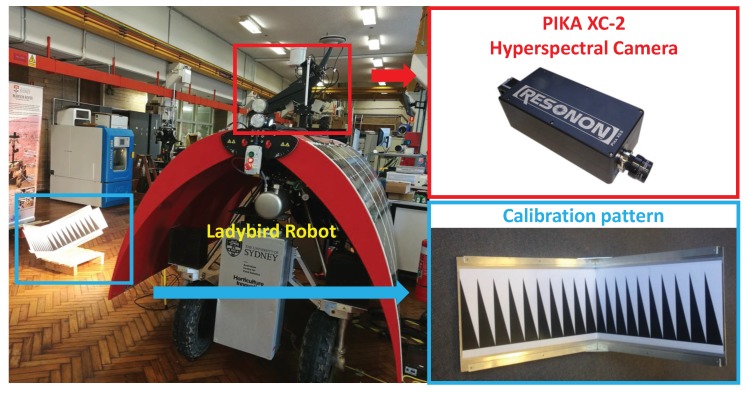
The experimental setup of the hyperspectral line-scan camera calibration.

In this section, calibration of a hyperspectral line-scan camera using real experimental data is presented. As shown in Figure 12, a hyperspectral line-scan camera was mounted on an agricultural robot, Ladybird. Ladybird was designed and built at the Australian Centre for Field Robotics (ACFR) at The University of Sydney as a flexible tool to support a range of agricultural research applications [28,29,30,31,32]. The Resonon Pika XC-2 visible to near infrared (VNIR) hyperspectral line-scan camera is mounted on top of the Ladybird platform and oriented such that the scan line is horizontal and pitched down for scanning the ground surface. The camera is configured to produce hyperspectral images of 1936 spatial by 1216 spectral pixels (a spectral resolution of approximately 1.3 nm from 400 to 1000 nm) at a frame rate of 100, 12-bit images per second. A Schneider Cinegon 8 mm lens is used, and manually focused with a checker board at the typical imaging distance. The parameters of the calibration target are the same as those used in the simulations and are summarized in Table 1.

There are 15 groups of calibration data recorded in the experiment, with each group of data recorded from one particular camera view angle. Each group of calibration data consists of 60 hyperspectral images, so there are 900 hyperspectral images all together for the calibration. The final calibration results of intrinsic parameters $f_{y}$, $v_{0}$, and $k_{1}$, together with the maximum re-projection error and the re-projection RMSE in pixels are shown in Table 2. The results are obtained using the joint nonlinear optimization using multiple camera poses. Each row in Table 2, corresponds to a calibration result where one group of data from a particular view angle is excluded. Since simulation results presented before show superior performance of the joint nonlinear optimization using multiple camera poses, the nonlinear optimization using single camera pose is not carried out.

Since there is no ground truth data available, no comparison is made with the conventional method [19]. However, by comparing the re-projection errors with the conventional method, we can see that both the maximum re-projection errors and the re-projection RMSE in all results in Table 2 are better than those reported in the conventional method, which has the maximum and re-projection RMSE of 1.779 and 0.547 pixel, respectively.

## 5. Discussion

The simulation and experimental results shown in Section 4.1 and Section 4.2 demonstrate that the proposed method can effectively estimate the calibration parameters using observations from multiple images and multiple view angles. The proposed joint optimization method yields better accuracy compared to the conventional method. There are several reasons that contribute to the performance of the joint optimization method.

A distinct advantage of the proposed joint optimisation method is that it can utilise observations from multiple images and multiple view angles in a principled manner. To take advantage of this feature, more images need to be recorded at different camera view angles. While this does increase the labour required to perform a calibration, the high frame-rate of line-scan cameras makes it easy to collect many images in a short time frame. The proposed signal processing step (Section 3.3) also eliminates the need for any labelling or manual feature extraction prior to optimisation. The experiment described in Section 4.2 only took around 15 min to record all 15 camera view angles. Since more parameters need to be optimised, the proposed joint optimisation method also needs more computing time. Despite needing more resources than the conventional method, the requirements are not onerous. The results reported in Section 4.2 took 10 min to generate using Matlab on a consumer grade desktop with frequency of 2.30 GHz per core in a single thread mode.

Another reason contributing to the better performance of the proposed joint optimization method is the way the calibration method is parameterised. As described in Section 3.5, in the conventional method, the least squares objective function is over-parameterized. This means the conventional method must update the intersection points $P_{i}$ at every optimisation iteration. This decoupling of correlated parameters leads to sub-optimal solutions since the resulting point $P_{i}$ are not guaranteed to be on the view plane defined by the resulting extrinsic parameters *R* and *T*. The proposed method removes the over-parameterisation. This is done by formulating the intersection points $P_{i}$ as functions of *R* and *T* so that the resulting solution is optimal for the problem defined.

A final significant improvement is that the optimization error function, Equation (31), has been redefined to reflect the re-projection errors of intersection points in pixels and to include the effect of radial distortion. The conventional method ignores radial distortion, which is known to be an important parameter [15]. In addition, in the conventional method, the residual error along the *X*-axis is not scaled to reflect the re-projection error in pixels as the residual error along the *Y*-axis is, which leads to different weights for errors along the *X*- and *Y*-axes.

The results shown in Figure 11 indicate that the calibration results are sensitive to the precision of the calibration target for both the proposed and conventional methods. For example, when the tilt angle between two planes of the calibration target has an error of 1 degree and the camera observation noise has a variance of 1 pixel, the estimation errors of focal length $f_{y}$ and principal point $v_{0}$ exceed 40 pixels. To make sure that errors in $f_{y}$ and $v_{0}$ are less than 20 pixels, the tilt angle should have no more than half a degree error. This enforces a strong tolerance requirement on the manufacturing of the calibration target. Another problem that can arise due to the sensitivity to the calibration target is, even if the target can be made with high precision, its shape can change slightly according to different temperature or as time goes by. One way to tackle this problem is to accurately measure the change (e.g., the tilt angle) before each time of calibration and update equations of 3D lines on the target accordingly.

The main sources of error, the observation noise and error in calibration target, have already been discussed. There are several other factors that can influence the accuracy of the result. The camera optical center is assumed to be aligned with the sensor array in both the proposed and conventional methods. However, when the optical center is not aligned with the center of the sensor and lens distortion is present, the view plane might not necessarily be straight in the world frame. In fact, the actual view plane might be slightly curved in this scenario, which means the camera model presented in Section 3.1 is not valid [15]. Another approximation made in the proposed method is that $f_{x}$ is substituted by $f_{y}$ in Equation (31). As mentioned, this is because $f_{x}$ is not needed for a line-scan camera calibration and is only used to scale the re-projection error ${\hat{x}}_{i}\left(R,T,f_{x},k_{1}\right)$ to proper pixel units. Since $f_{x}$ is almost the same as $f_{y}$ in terms of magnitude, $f_{y}$ can be used to scale the re-projection error ${\hat{x}}_{i}\left(R,T,f_{x},k_{1}\right)$ as well. This leads to very small different weights in re-projection errors along the *X*- and *Y*-axes, but the effect is marginal.

## 6. Conclusions

In this paper, we present an improved cross-ratio invariant based calibration method to calibrate the intrinsic parameters of a hyperspectral line-scan camera. As opposed to the conventional method [19], the calibration method proposed in this paper has been formulated to include the radial distortion coefficient of a camera lens. Another major contribution of the proposed approach is its ability to estimate the camera intrinsic parameters from multiple images gathered at multiple camera view angles. To facilitate this feature, we also present a signal processing method for converting images into calibration features. The result is a principled calibration frame work that can produce accurate estimates with little human supervision. Comprehensive simulation and experimental results show our proposed method is able to produce more accurate and consistent results compared to previous methods. Potential future work includes estimation of error parameters in the calibration target, such as the error in the angle between the orthogonal planes, together with the intrinsic parameters. This will further improve accuracy of the calibration and make the method more robust to the error in calibration target due to manufacturing tolerances.

## Figures and Tables

**Table 1:** Parameters in simulation.

Parameters	Values
Width of the triangle in the target ($w_{p}$)	0.24 m
Height of the triangle in the target ($h_{p}$)	0.04 m
Ground truth value of the focal length ($f_{y}$)	5000 pix
Ground truth value of the principal point ($v_{0}$)	1024 pix
Number of pixels in spatial dimension	2048
Rotation (*R*) of camera in axis angle	(−163.6, 0.095, 8.203)${}^{\circ }$
Translation (*T*) of camera	(−0.071, 0.115, 1.671) m
Calibration images for each camera view angle	100
Number of camera view angles	15
Least square optimiser	Levenberg–Marquardt

**Table 2:** Calibration results of the experimental data. The units of $f_{y}$, $v_{0}$, and re-proj errors are in pixels.

No.	Focal Length $f_{y}$	Principal Point $v_{0}$	Radial Distortion $k_{1}$	Max Re-Proj. Error	Re-Proj. RMSE
1	3922.7	989.0	−0.0110	1.7648	0.3782
2	3925.5	988.6	−0.0078	1.7684	0.3776
3	3926.4	988.3	−0.0062	1.7693	0.3764
4	3920.6	990.1	−0.0134	1.7640	0.3794
5	3910.8	990.1	−0.0239	1.7466	0.3804
6	3914.5	998.4	−0.0221	1.7762	0.3766
7	3914.3	998.4	−0.0234	1.7756	0.3756
8	3911.2	999.0	−0.0281	1.7708	0.3748
9	3919.0	995.6	−0.0149	1.7771	0.3783
10	3922.1	992.7	−0.0111	1.7062	0.3799
11	3918.9	990.0	−0.0144	1.7610	0.3764
12	3918.4	990.3	−0.0143	1.7609	0.3756
13	3916.2	992.1	−0.0165	1.7623	0.3743
14	3916.1	990.5	−0.0178	1.7574	0.3768
15	3915.5	991.3	−0.0189	1.7584	0.3782
Mean	3918.1	992.3425	−0.0163	1.7613	0.3772
STD	4.7088	3.7250	0.0062	0.0173	0.0018
